# Stripe noise removal in conductive atomic force microscopy

**DOI:** 10.1038/s41598-024-54094-w

**Published:** 2024-02-16

**Authors:** Mian Li, Jan Rieck, Beatriz Noheda, Jos B. T. M. Roerdink, Michael H. F. Wilkinson

**Affiliations:** 1 Bernoulli Institute for Mathematics, Computer Science and Artificial Intelligence, University of Groningen, Groningen, The Netherlands; 2 Zernike Institute for Advanced Materials, University of Groningen, Groningen, The Netherlands

**Keywords:** Atomic force microscopy, Image processing, Computer science

## Abstract

Conductive atomic force microscopy (c-AFM) can provide simultaneous maps of the topography and electrical current flow through materials with high spatial resolution and it is playing an increasingly important role in the characterization of novel materials that are being investigated for novel memory devices. However, noise in the form of stripe features often appear in c-AFM images, challenging the quantitative analysis of conduction or topographical information. To remove stripe noise without losing interesting information, as many as sixteen destriping methods are investigated in this paper, including three additional models that we propose based on the stripes characteristics, and thirteen state-of-the-art destriping methods. We have also designed a gradient stripe noise model and obtained a ground truth dataset consisting of 800 images, generated by rotating and cropping a clean image, and created a noisy image dataset by adding random intensities of simulated noise to the ground truth dataset. In addition to comparing the results of the stripe noise removal visually, we performed a quantitative image quality comparison using simulated datasets and 100 images with very different strengths of simulated noise. All results show that the Low-Rank Recovery method has the best performance and robustness for removing gradient stripe noise without losing useful information. Furthermore, a detailed performance comparison of Polynomial fitting and Low-Rank Recovery at different levels of real noise is presented.

## Introduction

**Figure 1:**
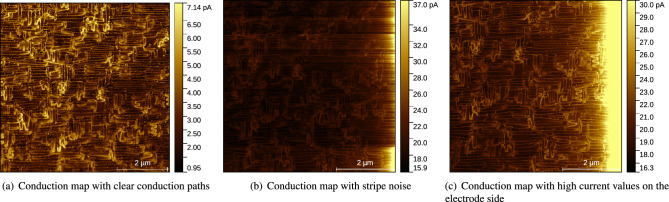
Three conduction maps of the same sample collected by lateral measurement, where the fixed electrode is at the right side. The conduction paths in (**b**) and the right edge of (**c**) are less clear than in (**a**) because of the stripe noise and the high current values at the edge of the electrode side, respectively.

In recent years, conductive atomic force microscopy (c-AFM) has been widely used for imaging local conductivity in materials^1–3^ and is playing an increasingly important role in the optimization of materials for their use in novel microelectronic devices, including ferroelectric tunnel junctions^4^, resistive switching memories^5^ and memristors^6^. This technique can be extremely useful, ranging from detecting defects of a couple of atoms in size to measuring the different resistance states of a memory. In addition, c-AFM has been key to investigating conduction paths in multiferroic materials. These are self-organized networks of topological defects that form periodic patterns and can carry electrical currents, acting as a dense mesh of nanoscale conducting paths^7^ and are believed to hold promise for future electronic devices^8^. To explore the properties of conduction paths in the samples, see Fig. 1a as an example, one needs to identify and extract these paths in the conduction maps, which are mainly collected by c-AFM^7,9,10^. The metallic tip of the c-AFM’s cantilever (with an end diameter of about 20nm), acting as an electrode, is brought in contact with the sample surface and it applies a voltage at that location by means of an electrical circuit, which also returns the electrical current measured across the sample (vertically, if the second electrode is located below the sample). After scanning all the points of the sample surface within the scan area, a conduction map is produced, from the local values and variations of which the conductivity/resistivity of the materials can be inferred.

However, many artifacts, mainly stripe noise, occur in c-AFM measurements, especially in lateral measurements. Compared with vertical measurements, where charge flows from the top to the bottom electrode, lateral measurements^10^ use a second (fixed) top electrode to achieve a charge flow parallel to the surface, between the fixed electrode and the scanning tip. Therefore, the generation of stripes could be related to charge accumulation and drift, since the direction of this stripe noise also coincides with the direction of the current from the electrode side. There are three conduction maps measured laterally on the same sample in the experiments of Rieck et al.^10^, where the fixed electrode side is at the right side in Fig. 1. The conduction paths in the conduction map (a) are clearly visible as the measuring area is only a few micrometres away from the electrode edge. The measuring area of (b) and (c) is almost the same, but at the edge of the electrode side there is a lower quality of conduction paths, as very high currents are generated as soon as the conductive tip touches the electrodes at this voltage. In (b), the stripes cross the entire image, and change values as they pass through the higher-conductivity paths (conduction paths). Due to the lateral electrode geometry used, the stripe noise in the image shows an intensity gradient that matches the current direction from the fixed electrode (which occupies the entire right side of the image) to the tip. Image (c) appears to be cleaner, but the current values on the electrode side are higher and display some glitches, so the conduction paths at the edge are not as clear as in (a). It is therefore possible that the extremely strong potential differences lead to stripe noise. There may be another cause for the occurrence of stripes that is not related to conductivity, since c-AFM measures not only the electrical current flow but also the topography at the tip contact point at the same time. A main cause of the stripe noise in c-AFM is due to a changing tip-sample interface, which could be caused by contaminants such as dirt particles adhering to the tip, or irregular edges or protrusions on the sample surface^11^, or by “parachuting” artifacts caused by high scanning speed^12,13^. The latter is not very likely in our case, as scanning speeds are low.

Finding the most robust and effective method to remove the stripes is important in c-AFM image processing. Stripe noise significantly impairs the observation and hinders subsequent analysis of conduction components. Moreover, the difficulty in obtaining clean images caused by the high sensitivity of the collection and the challenging experimental conditions means that we have to make better use of the existing noisy c-AFM images by applying denoising methods. Finally, analyzing stripe noise removal methods could help in investigating the physical source of the noise, which would enable us to find more effective removal methods. We may also be able to find correlations between some scan parameters and the noise model^13,14^. Adjusting the scan parameters can help to reduce noise generation during scanning.

Among the most advanced or commonly used methods for removing stripe noise from AFM images are Destripe2^15^, VSNR^16^ and algorithms from the tool Gwyddion^14,17^, which have proven to be effective in removing stripe noise from scanning electron microscope modalities. Gwyddion is an important and popular modular program for processing and visualize AFM images. This makes Gwyddion the first choice for surface physicists when it comes to image processing tasks that involve denoising the images they measure. The work on denoising AFM images is usually compared with the results of Gwyddion’s algorithms^15,16^.

Another method worth discussing but not used in the field of AFM image processing is SNRWDNN (Stripe Noise Removal Wavelet Deep Neural Network)^18^, which was developed for stripe noise removal only. In this method a directional regularizer function is designed to separate the details of the scene from the stripe noise and prevent irregular stripes in the estimation of the clean image. This means that this Deep Learning model could work very well in our particular case of gradient stripe noise, even though it was not developed specifically for AFM images.

Finding a good solution to remove stripe noise is a time-consuming and challenging task for the scanning probe microscopy user community. Even though the number of open-source software tools like Gwyddion is limited, it would be a time-consuming task to try out all of the denoising functions of the c-AFM images processing software tools and the state-of-art c-AFM image denoising algorithms, which is, indeed, not typically done by surface physicists.

There are many robust methods that are not yet used in the field of AFM image processing, but have already proven to be effective and are well developed in other applications for stripe noise removal. These include frequency-based algorithms, statistics-based methods, polynomial fitting algorithms, and difference correction algorithms. As the noise intensity varies, the optimal destriping methods change accordingly. To handle this issue we may consider optimization-based methods. For example, in the field of remote sensing, Group Sparsity-based Regularization (GSR) and Unidirectional Total Variation (UTV) as well as low-rank matrix recovery-based methods have been developed and fully compared^19,20^. These classical and effective optimization-based methods are not yet used for processing noise in AFM images.

Therefore, the field of c-AFM processing is in great need for a review of destriping methods and extensive experimentation in order to provide the most reliable methods and tools, and give recommendations for the use of image processing methods that are very effective but have not been used in c-AFM image processing so far.

In this study, we compare a total of 16 different artefact removal algorithms for c-AFM conduction maps. These include 13 state-of-the-art destriping methods and three additional optimization-based methods that we tailored towards the characteristics of stripe noise. After extensive experiments on natural and simulated noisy images, we determined the best processing method.

The rest of the paper is structured as follows. In Sect. ‘Methods’, we present three different assumptions and corresponding models based on the stripe noise characteristics. Extensive experiments are presented and discussed in Sect. ‘Experiments’, including qualitative and quantitative visual comparisons of image quality. In the qualitative comparison, we first evaluate the 16 different methods by comparing the destriping results on a natural noisy image. Next, we compare all methods by computing destriping results of simulated images generated by our designed noise model to evaluate the consistency. For this purpose, simulated noise was added to a ground truth image to enable a quantitative comparison of image quality. To comprehensively test the robustness of the 16 methods, two types of experiments were conducted: (1) a simulated image dataset with the noise closest to the real noise, and (2) a series of images with very different noise intensities In Sect. ‘Discussion’, we discuss the experimental results, including computing time, machine requirements and required parameters, as well as the design of the methods. Finally, we summarize this study and suggest future work in Sect. ‘Conclusion’.

## Methods

In this section, we make three different assumptions on the nature of the stripe noise and the clean image, and introduce three recovery models involving the observed image, the clean image and the noise.

Before presenting our assumptions let us make some observations. We can see very clear conduction paths in Fig. 1a. The conduction paths are the bright structures in the image, some of which are meandering and twisted, and some are connected in rows. However, in Fig. 1b we cannot discern the conduction paths clearly because of the stripe noise. The model assumptions for removing these are as follows:

1. *The noise is low rank* we see that the stripes appear as a series of closely spaced, parallel vertical lines extending from right to left, with different intensity and thickness, but similar properties. The rank of this noise pattern would be relatively low, as there are only a few distinct patterns. This means that the noise can be decomposed into a small number of independent patterns. It appears as relatively uniform and closely spaced stripes, while the main features of the conduction paths remain recognisable.
2. *The noise is group sparse* there are certain areas or regions of the observed image that are affected by the stripes, while other regions are unaffected or have minimal noise. Also, the noise manifests itself as groups that occur in specific patterns with varying density, spacing, and thickness, rather than uniformly affecting the entire image, so that we can visually distinguish it from the surrounding clean regions.
3. *The clean image has minimal unidirectional total variation* in the observation image, the stripe noise causes abrupt transitions and discontinuities in the horizontal direction, increasing the UTV of the image. So we can use total-variation regularization, a popular method for image denoising. Total-variation regularization minimizes the UTV of the image by promoting sparsity in the gradient or edge information. By applying this regularization, the algorithm could effectively remove the stripes while preserving the edges and important features of the conduction paths. Here we introduce the possibility of horizontal and vertical directions with minimal UTV.

Based on these three assumptions, we present three classical models that have never been used for denoising AFM images. The models are described below.

### Low-rank recovery

In image destriping via low-rank recovery (LRR)^21^, the observed images are modeled as the sum of a clean image and stripe noise which is of low rank. Let *N* denote the observed image, *M* the clean image, and *L* the low-rank stripe noise. That is, assume that:

1 $$N=M+L$$

Image destriping via LRR is performed as follows: first, obtain an estimate $L^*$ of the low-rank stripe noise from the observation *N*, then obtain an estimate $M^*$ of the clean image via

2 $$\begin{aligned} M^*=N-L^*. \end{aligned}$$

The stripe-noise estimate $L^*$ is computed via an optimization problem as follows:

3 $$\begin{aligned} L^{*}=\arg \min _{\Lambda } \frac{1}{2}\Vert \Lambda -N\Vert _{2}^{2}+\lambda \Vert \Lambda \Vert _{*} \end{aligned}$$

where the notation $||\Lambda \Vert _{*}$ denotes the nuclear norm of $\Lambda$, i.e., the sum of the singular values of $\Lambda$, and the regularization parameter $\lambda$ controls the trade-off between the two objectives of fitting and low-rank regularization, respectively.

The optimization problem Eq. (3) can be transformed into a well-known form via singular value decomposition. That is, suppose the singular value decompositions of $L^*$ and *N*, respectively, are as follows:

4 $$\begin{aligned} L^*=U\,S^*\, V^T \end{aligned}$$

and

5 $$\begin{aligned} N=U\,S\,V^T \end{aligned}$$

Then the optimization problem Eq. (3) is equivalent to:

6 $$\begin{aligned} S^{*}=\arg \min _{\Lambda } \frac{1}{2}\Vert \Lambda -S\Vert _{2}^{2}+\lambda \Vert \Lambda \Vert _{1} \end{aligned}$$

It is worth noting that the norm of the regularization term in Eq. (6) has become the $l_1$ norm, as compared with the nuclear norm in Eq. (3). Hence, Eq. (6) is the well-known shrinkage or soft-thresholding formulation, of which the closed-form solution is as follows: the entry (*i*, *j*) of $S^*$ is given by

7 $$\begin{aligned} S_{i, j}^{*}={\text {sign}}\left( S_{i, j}\right) \max \left\{ S_{i, j}-\lambda , 0\right\} \end{aligned}$$

In conclusion, the method of destriping via LRR is performed as follows. Given an observed image *N* and a regularization parameter $\lambda$:

1. compute the singular value decomposition of the observation *N*, as in Eq. (5), obtaining its singular value matrix *S*;
2. perform the soft-thresholding operation on the matrix *S* with the parameter $\lambda$ via Eq. (7);
3. recover the low-rank stripe noise estimate $L^*$ via Eq. (4);
4. compute the clean-image estimate *M* via Eq. (2).

### Group sparse recovery

In destriping via group sparse recovery (GSR)^22^, the observed image is modeled as the sum of the clean image and stripe noise that is group sparse or column sparse. Suppose *N* denotes the observed image, *M* the clean image, and *G* the group sparse stripe noise, then we assume that:

8 $$\begin{aligned} N=M+G. \end{aligned}$$

Image destriping via GSR is performed as follows: first recover the group sparse stripe noise estimate $G^*$ from the observation *N*, then obtain an estimate $M^*$ of the clean image via

9 $$\begin{aligned} M^*=N-G^*. \end{aligned}$$

The estimate $G^*$ of the stripe noise is recovered via an optimization problem as follows:

10 $$\begin{aligned} G^*=\arg \min _{\Gamma } \frac{1}{2}\Vert \Gamma -N\Vert _{2}^{2}+\mu \Vert \Gamma \Vert _{2,1}, \end{aligned}$$

where the notation $||\Gamma \Vert |_{2,1}$ denotes the $\ell _{2,1}$ norm of $\Gamma$, i.e.,

11 $$\begin{aligned} \Vert \Gamma \Vert _{2,1}=\sum _{j=1}^{n}\left\| \Gamma _{j}\right\| _{2}. \end{aligned}$$

Here $\Gamma _j$ denotes the $j^{th}$ column vector of the matrix $\Gamma$, and the regularization parameter $\mu$ controls the trade-off between the two objectives of fitting and group sparse regularization.

The optimization problem Eq. (10) has a closed form solution^23^, which is computed as follows: the $j^{th}$ column vector of the matrix $G^*$ is given by:

12 $$\begin{aligned} G_{j}^{*}=\frac{N_{j}}{\left\| N_{j}\right\| _{2}} \max \left\{ \left\| N_{j}\right\| _{2}-\mu , 0\right\} . \end{aligned}$$

In conclusion, the method of destriping via GSR is performed as follows. Given an observation image N and a regularization parameter $\mu$:

1. recover the group sparse stripe image estimate $G^*$ with the parameter $\mu$ via Eq. (12);
2. compute the clean image estimate $M^*$ via Eq. (9).

### Unidirectional total variation minimization

When destriping via UTV minimization^24^, the observed image is modeled as the sum of the clean image, which is supposed to have minimum UTV, and stripe noise. Suppose *N* denotes the observed image, *M* the clean image, and *L* the noise, then we assume that:

13 $$\begin{aligned} N=M+L \end{aligned}$$

The clean image estimate $M^*$ is obtained via an optimization problem as follows:

14 $$\begin{aligned} M^{*}=\arg \min _{\mathscr {M}} \frac{1}{2}\Vert \mathscr {M}-N\Vert _{2}^{2}+\nu \Vert \mathscr {M}\Vert _{U T V} \end{aligned}$$

where the notation $\Vert \mathscr {M}\Vert _{U T V}$ denotes the unidirectional total variation of $\mathscr {M}$. The discrete forms of horizontal and vertical UTV (denoted as $UTV_1$ and $UTV_2$) are defined as

15 $$\begin{aligned} \Vert \mathscr {M}\Vert _{U T V_1}=\sum _{i=1}^{m} \sum _{j=1}^{n}\left| \mathscr {M}_{i, j}-\mathscr {M}_{i, j+1}\right| \quad \quad \text {and} \quad \quad \Vert \mathscr {M}\Vert _{U T V_2}=\sum _{i=1}^{m} \sum _{j=1}^{n}\left| {\mathscr {M}}_{i, j}-\mathscr {M}_{i+1, j}\right| , \end{aligned}$$

respectively. These models are modifications of classic total variation minimization (also known as the Rudin-Osher-Fatemi model):

16 $$\begin{aligned} \mathscr {M}=\arg \min _{\mathscr {M}} \frac{1}{2}\Vert \mathscr {M}-N\Vert _{2}^{2}+\nu \Vert \mathscr {M}\Vert _{T V}, \end{aligned}$$

and total variation

17 $$\begin{aligned} \Vert \mathscr {M}\Vert _{T V}=\iint _{\Omega }\left[ \left( \frac{\partial \mathscr {M}}{\partial x}\right) ^{2}+\left( \frac{\partial \mathscr {M}}{\partial y}\right) ^{2}\right] ^{1 / 2} \mathrm {~d} x \mathrm {~d} y. \end{aligned}$$

The modification is based on the observation that stripe noise has only one direction.

## Experiments

To find the most robust and efficient method for removing stripe noise from c-AFM images, we developed a noise model and performed intensive comparisons on a noisy c-AFM image and simulated noisy images. All the conduction maps in this paper are from the experiments on the same sample reported by Rieck et al.^10^.

The comparison of the 16 selected methods includes (1) the three models from the last section, (2) all denoising methods using line-by-line scanning in Gwyddion, (3) a deep learning method developed and trained for stripe noise removal only, and (4) two state-of-the-art denoising methods developed for AFM images. This section is divided into three subsections: "Method comparison", "Visual comparison", and "Quantitative image quality comparison".

In the Method Comparison subsection, we briefly explain each method and the reasons why we selected it, and propose the noise model. The next subsection concerns the visual comparison of the destriping image results. We analyze the results of natural and simulated noise removal in the Sect. "Quantitative image quality comparison", using SSIM (Structural Similarity Index)^25^ and PSNR (Peak Signal to Noise Ratio)^26^. The first experiment uses an image dataset with fixed noise weights. In this experiment, we create a dataset of 800 ground truths by flipping and cropping a clean image and adding random simulated stripe noise to obtain a corresponding dataset of 800 simulated noisy images. The second "Quantitative image quality comparison" experiment uses a set of images that have very different noise weights. We obtain boxplots of the 800 PSNR and SSIM results from the first experiment, and SSIM and PSNR curves from the second experiment. both leading to the same conclusion regarding the best denoising method.

### Method comparison

In this subsection, we briefly describe each method and the reasons why we included it in the comparison.

#### Methods from the SPM image processing tool

The Scan Line Artefacts functions in Gwyddion^14^ are used to flag and correct for various artefacts in AFM data related to line-by-line acquisition. It is important to include these functions for eliminating stripe noise caused by line-by-line scanning in our comparison. The 9 different Scan Line Artefacts algorithms in Gwyddion include statistical correction algorithms (finding a representative statistic for each scanline such as Median, Mode, and Trimmed Mean, and then subtracting it from the corresponding scanline); difference correction algorithms (Median Difference, Matching, Trimmed Mean Difference and Facet Level Tilt); a Polynomial Fitting Algorithm (which is mentioned in Section ’Introduction’ as the best method to remove leveling artifacts); and a Defect Marking Algorithm (based on user-defined criteria).

#### Methods for microscopic image processing only

We add VSNR (Variational Stationary Noise Remover)^16^ and DeStripe^15^ in our comparison. VSNR uses a simple noise model and solves a convex programming problem through numerical optimization. It has shown improved image quality in various denoising applications, including microscopic imaging modalities. DeStripe is an advanced denoising protocol developed for AFM biomolecular imaging. It effectively removes stripe noise by applying a divide-and-conquer approach in the frequency domain, preserving edge sharpness and enhancing molecular feature visualization, leading to better interpretation of AFM images.

#### Deep learning model developed only for stripe noise removal

Unlike other existing destriping methods, the deep learning model SNRWDNN^18^ was developed only for denoising stripes using the HDWT (Horizontal Discrete Wavelet Transform) to represent the directional properties of the strip components. It uses complementary information between different sub-bands to predict the strength and distribution of the noise. A directional regularizer function was designed to separate the scene details from stripe noise and to prevent irregular stripes in the estimation. This means that this deep learning model could perform very well in our gradient case. Therefore, we include it for comparison in our experiments.

We use the source code of the current stable version Gwyddion (version 2.63, Code release time: 2023-06-13) and use Pygwy to bind Gwyddion’s objects, methods and functions to write our destriping comparison modules.

Further details on the methods used in our comparison can be found in Table 1 with the abbreviations used in this work. Below, Median Difference, Facet Level Tilt, Polynomial, Trimmed Mean, Trimmed Mean Difference, Remove Scar, SNRWDNN and Destripe2 are abbreviated as MD, FLT, Poly, TM, TMD, RS, WNN (Wavelets Neural Network) and DS2. UTV1 and UTV2 denote vertical UTV and horizontal UTV, respectively.

**Table 1:** Methods used for comparison.

Methods	Abbreviations	Parameters	Computing Time	Code Release Time	Environment
Matching	Matching	Scanning direction (the first scanning axis)	0.008	2022-11-03	C++ Python Python 2.7 GTK 2.0 PyGObject 1.8 PyCairo 1.8 PyGObject 2.1 Pygwy
Median	Median	Scanning direction	0.004		
Median Difference	MD	Scanning direction	0.016		
Modus	Modus	Scanning direction	0.006		
Polynomial	Poly	Scanning direction Order	0.086		
Remove Scar	RS	Scanning direction	0.021		
Trimmed Mean	TM	Scanning direction fraction	0.004		
Trimmed Mean Difference	TMD	Scanning direction fraction	0.005		
Facet Level Tilt	FLT	Scanning direction	0.005		
VSNR	VSNR	indexes of p-norms data terms regularization of TV-norm stopping criterion ball-diameter Maximal number of iterations	2.922	2011-08-09	Matlab
SNRWDNN	WNN	The weights for stripe noise	0.011	Jul 5, 2022	Python PyWavelets Pillow Tensorflow Keras
DeStripe2	DS2	pixel numbers in X &Y axes Real physical sizes multiplication or scale factor the pixel value	0.002	2012-05-12	Fortran 77 C
GSR	GSR	Scanning direction,$\lambda$	0.001	Written by ourselves	Matlab
UTV(vertical)	UTV1	$\lambda$	0.080		
UTV(horizontal)	UTV2		0.080		
LRR	LRR		0.053		

#### Noise model

A noise model is designed for generating noisy images that are quite similar to the natural stripe noise in c-AFM images. By comparing the natural noisy image in Fig. 1b and the conduction map with clear conduction paths in Fig. 1a, it can be seen that the stripe noise is the strongest at the right, and then fades fast within at a short distance and seems to disappear almost completely near the edge. According to these noise characteristics, the gradient noise model can be defined as:

18 $$\begin{aligned} L = \mathscr {G} *[A(x, y)\cdot e^{bx}+c], \end{aligned}$$

where *A* is group-sparse noise, $\mathscr {G}$ represents a Gaussian kernel and $*$ represents a 2D convolution operation, *b* and *c* are parameters whose optimal values are found by fitting one random column of the real noise.

Simulated noise with different weights is generated and added to the ground truth Fig. 1a. So the simulated image can be defined as:

19 $$\begin{aligned} {Simulated\,Noisy\,Image} = {Simulated\,Noise} *{Noise\,Weight} + {Ground\,Truth} \end{aligned}$$

**Figure 2:**
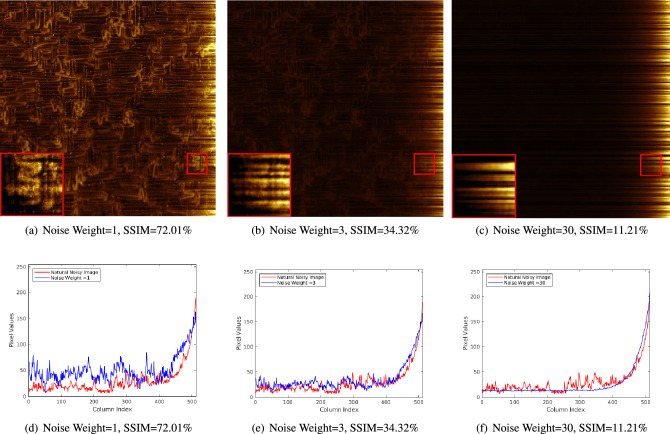
(**a**–**c**) Noisy images created by adding noise of different weights to the same ground truth (image in Fig. 1a); the corresponding SSIM values are indicated. The larger frames in the lower left corners of the images contain three times larger and contrast-enhanced versions of the contents of the small red frames at the same position in the center. The black pixel value curves in (**d**–**f**) represent a random column in the simulated images (**a**–**c**) (blue curves); in each plot the pixel value curve (in red) of the corresponding column in the natural noisy image of Fig. 1b is added.

Figure 2a–c are three simulated images generated by Eqs. (18) and (19). In (e), the red curve and the blue curve fit very well, which are two random columns of pixel values from image (b) and the natural noisy image Fig. 1b, respectively. This means that the noise model Eq. (18) and the noise level of (b) match well with the real noise. In the "Visual comparison" section, we use Fig. 2b as the image for the denoising comparison because the curves and markers show that it is very close to the real noisy image.

From (a)–(c) and their red frames, we can see that as the noise weighting increases, the conduction paths becomes less and less clear.

We used PSNR and SSIM, two commonly used metrics to quantify the correspondence between images. PSNR focuses on the signal *differences* while SSIM focuses on the *similarity* between the original and distorted images. In this work, we focus mostly on the SSIM value in the analysis of the denoising effect, while PSNR is only used as an auxiliary analysis. SSIM provides a more meaningful assessment of image quality and better fulfills our goal of detecting conduction paths.

The SSIM index for quality assessment is based on the calculation of three factors: luminance (*l*), contrast (*c*) and structure (*s*). The overall index is a multiplicative combination of these three factors:

20 $$\begin{aligned} SSIM(x, y) = [l(x,y)]^a\times [c(x,y)]^\beta \times [s(x,y)]^\gamma \end{aligned}$$

where *l*, *c* and *s* are calculated by the local means and standard deviations for images *x* and *y*, respectively. Using the common assumptions, the equation simplifies to:

21 $$\begin{aligned} SSIM(x,y)=\frac{(2\mu _x\mu _y+C_1)(2\sigma _xy+C_2)}{(\mu ^2_x+\mu ^2_y+C_1)(\sigma ^2_x+\sigma ^2_y+C_2)} \end{aligned}$$

where $\mu _x$, $\mu _y$, $\sigma _x$,$\sigma _y$ are the local mean values and standard deviations for the images *x*, *y*, respectively. SSIM values are between 0 and 1, where 1 means a perfect match between the two images that are compared (we express SSIM values as percentages below).

### Visual comparison

In this subsection, we perform a visual comparison of the 16 methods for removing noise on the natural noisy image in Fig. 1b and a simulated image.

**Figure 3:**
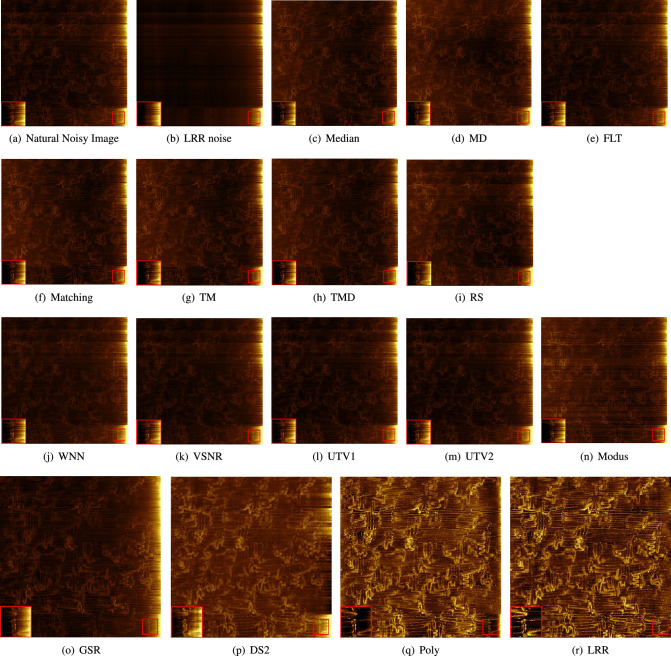
Image results of removing natural stripe noise: (**a**) is the natural noisy image (identical to Fig. 1b). (**b**) is the noise result of LRR. (**c**-**r**) are the denoising results of the 16 methods. (**o**–**r**) are enlarged images denoised by the four best methods, where the conduction paths can be seen in detail. The abbreviations of the methods are listed in Table 1. The larger frames in the bottom left corners of the images contain double-sized and contrast-enhanced versions of the contents of the small red frames at the bottom right corners (Same in Fig. 4).

#### Experiments with a natural noisy image

The results for the natural noisy image of Fig. 1b are shown in Fig. 3. In Fig. 3c–r, which contain the denoising results of the 16 methods.

Depending on the least remaining noise that can be seen, the four best methods are selected and enlarged in (o), (r), which allow us to look at the conduction paths in detail to make further observations. Only LRR, Poly, DS2 and GSR are able to show the texture of the conduction path more clearly. LRR and Poly are the only two that remove most of the stripes while recovering most of the conduction path. For the red frames in (q) and (r), LRR is better as more details of the path are recovered at the edge. In Fig. 3o, we can see that GSR removes some conduction paths but some stripe noise remains. While LRR perfectly removes all the stripe noise and restores the whole conduction paths, GSR, DS2 and Poly remove most of the noise and preserve most of the conduction paths.

The images of most other methods (c)–(n) are still as dark as the original images and not many stripes are removed. Although the result of Modus (e) slightly brightens the conduction path, there are some streaks across the entire image.

#### Experiments with a simulated noisy image

**Figure 4:**
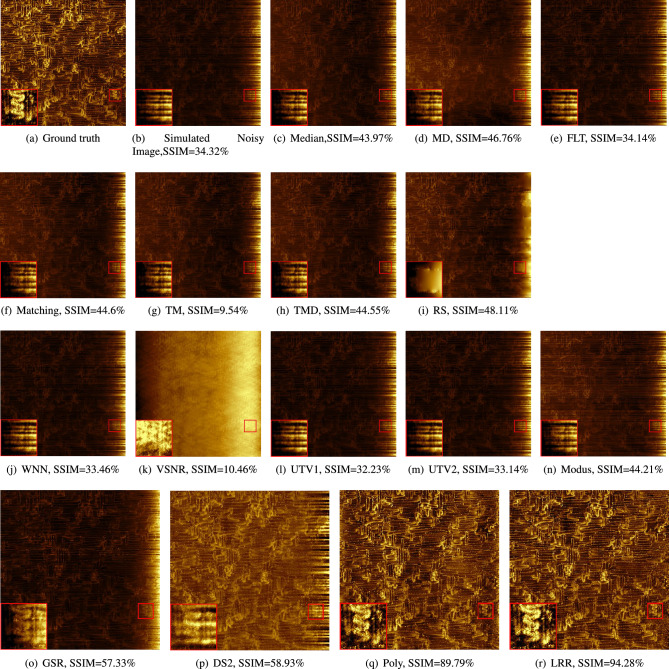
Results of removing simulated stripe noise: (**a**) is the ground truth. (**b**) is the simulated image after adding simulated noise to the ground truth (**a**). (**c**–**r**) are the denoising results of the 16 methods. (**o**–**r**) are enlarged images denoised by the four best methods, where the conduction paths can be seen in detail.

We used our noise model to generate noisy images for the second experiment. The denoising results for the simulated images are shown in Fig. 4. Except VSNR, the image denoising results and SSIM values in Fig. 4 are consistent with the denoising results of the natural image in Fig. 3: LRR, Poly DS2 and GSR are the best four methods, with LRR it the best. The VSNR result (l) looks different because we used a different input filter in the experiment with denoising the natural noisy image. This is explained under Parameter settings in section ’Discussion’. We see that LRR is the only method whose SSIM score is above 90%. The SSIM value of Poly is close to 90%, and DS2 and GSR more than double the SSIM value of image (b). The images denoised by FLT, WNN, VSNR, UTV1 and UTV2 have the lowest SSIM values. Most other methods also have the same performance in Fig. 3, which are dark and also the result of Modus is the same: brighter, but some stripes cross the image result. The image results in Fig. 4 are comparable with the those in Fig. 3 (except for VSNR (l)).

### Quantitative image quality comparison

There are two types of experiments in this subsection: (1) experiments with a large dataset of simulated noisy images that have a similar SSIM value as the real noisy image; and (2) experiments with simulated noisy images with 100 different noise levels, which allow us to evaluate the robustness of the methods at different noise levels.

#### Experiments with the simulated noisy dataset

**Figure 5:**
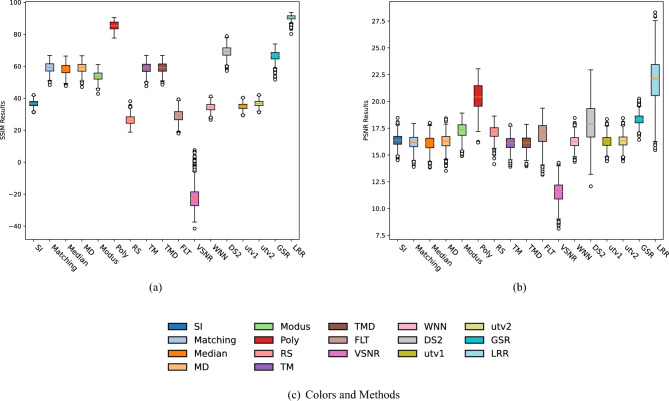
Box plots for SSIM and PSNR results for the 16 denoising methods on the simulated dataset (800 image pairs).

Each image in the simulated noisy dataset has a similar SSIM value as the natural noisy image, obtained by manipulating the noise weights. There are a total of 800 pairs of ground truth images and corresponding simulated noisy images with an average SSIM value of 36.57. Figure 5 shows the SSIM and PSNR boxplots corresponding to the denoising results and their ground truths.

The average SSIM values of GSR, DS2, Poly and LRR in Fig. 5a are the highest and are 66.42%, 69.23%, 85.5% and 90.43%, respectively. UTV1, FLT, RS, VSNR have the lowest of 34.82%, 29.18%, 26.37%, 21.49%. The SSIM boxes of all methods in (a) are relatively short, which means that each method gives comparable results for the images in the dataset. We can easily interpret the SSIM results as consistent with the visual comparison by comparing the average values. As we can see from Fig. 5b, LRR outperforms the other methods also when using the PSNR metric. In (a), the Poly method ranks second to LRR, and is much better than the other methods.

#### Experiments with simulated images of different noise strengths

**Figure 6:**
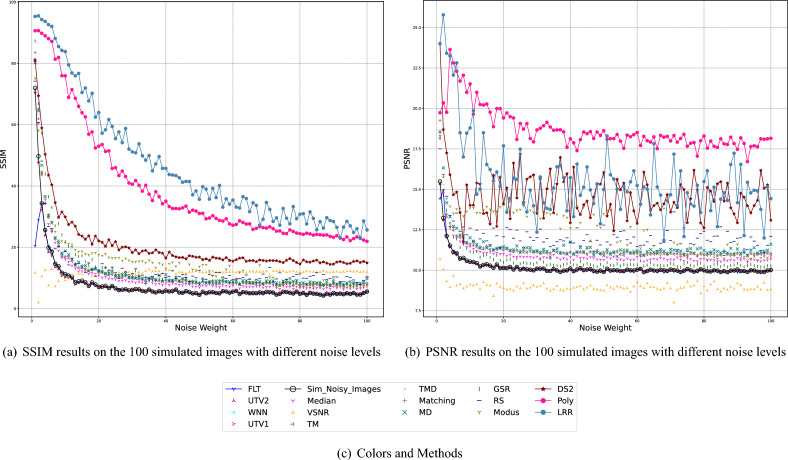
Experimental results for images with variable noise weights. (**a**, **b**) SSIM and PSNR curves between the denoised images and the ground truth image for the 16 denoising methods. The black curve with circles represent the SSIM and PSNR values between the simulated images and the ground truth image.

To discuss more general denoising situations, 100 images with 100 distinct noise levels are simulated and denoised, varying the noise weight from 0 to 100. The SSIM range of the resulting dataset is from 5.58% to 72.01%. The results are shown in Fig. 6, in which the curves show the SSIM and PSNR curves between the denoised images and the ground truth image for the 16 denoising methods.

From the SSIM curves, we can see that most of the results are consistent with the conclusions from the visual comparison and the experiment with the simulated noisy dataset. The best methods and the worst methods are almost the same.

In the PSNR results, LRR performs below Poly at most noise levels. This is probably due to the fact that LRR tends to brighten the image. SSIM is less sensitive to this. Although Figure 3 shows that the Poly result is darker than the LRR result, the results are opposite for most of the different noise levels. The noise weight of Fig. 3 is 3 and we can see in Fig. 6b that the LRR PSNR value is still higher than that of Poly because the LRR result is brighter than Poly. Most of the remaining image results with higher noise weight are brighter with Poly than with LRR, but the structure of the conduction paths in these Poly results is not as obvious as in the LRR results. For this reason, the SSIM and PSNR results of LRR and Poly are inconsistent.

## Discussion

We first summarize the results of all the experiments in this paper, in particular the comparisons between the denoising results of LRR and Poly. Then we compare all methods in-depth based on the method designs, computational efficiency (computation time and machine requirements), and input parameters. For the quantitative results, we mainly look at SSIM in this section.

### Results analysis

Firstly, the results of the different comparisons, whether visual or quantitative, in this paper are generally consistent. LRR has the best overall performance for real and similar simulated noisy images in terms of SSIM. Poly is a fairly close second, and in terms of PSNR, it outperforms LRR in Fig. 6b. Overall, the performances of these two methods are quite similar. The results shown in Fig. 2 showed that our noise model matches the real noise well.

### Computational efficiency

In terms of computational efficiency, as we can see in Table 1, the computing times of almost all methods are short, that is, less than 0.1 seconds for the $520\times 520$ natural image, except for the VSNR method which took 2.92 seconds. Note that the WNN method requires a GPU for fast performance.

### Method designs

In the "Method" Section, LRR and GSR were proposed as denoising models for processing c-AFM images based on the data redundancy features of stripe noise. Of the two, LRR is apparently more effective in removing the stripe noise.

In previous work, polynomials were among the best methods for removing gradient stripe noise, but our new results show that LRR generally has an edge.

DS2 was specially developed for the removal of stripe noise in AFM images. Most results show that DS2 ranks third among the 16 methods. DS2 requires three topographic measurement parameters as input for the frequency information to enhance the features. The visual comparison showed that DS2 performs well, but is inferior to LRR and Poly, as the denoised images still show some strong stripes.

### Parameter settings

The input weights for WNN have been trained for stripe noise so that no parameter needs to be input manually. Most other methods require one or more parameters of the sample, which can be determined by trial and error. UTV and LRR require only a single input parameter $\lambda$, which was fixed in all experiments. An optimal value of $\lambda$ was found by trial and error in the first comparison. As we can see in Table 1, all methods of Gwyddion and GSR require the scan direction. Poly, TM and TMD only require one additional parameter.

VSNR does not provide a specific method for determining the optimal parameters for the input. However, it is mentioned that the filters used in the algorithm are selected based on the application and the type of noise present in the image^16^. In this work, we directly used a column of stripe noise as a filter and compared the performance of the VSNR algorithm with other methods as shown in the paper. It is possible that the authors used a trial-and-error approach to find suitable parameters for their specific images and noise types, but finding the optimal parameters for the VSNR algorithm can be time consuming. Compared to the other methods VSNR is more time consuming and requires more parameters.

For most comparison methods, we used the optimal or correct parameter values that we determined experimentally.

### Summary

Based on the discussions above on the comparative results, computational efficiency, and input parameters, we conclude that LRR is the most suitable method for removing gradient stripe noise and retaining the original features in c-AFM images, closely followed by Poly. It has the most competitive results, is relatively fast without needing a GPU, and requires only one parameter, which is fixed and works well for all comparisons.

## Conclusion

To preserve the structure of the conduction paths in c-AFM images, we have compared 16 methods for removing the stripe noise that occurs in lateral measurements of ferroelastic oxide materials.

First, we verified the assumption that the stripe noise in lateral measurements is low rank and found that LRR is the best method to remove it, with Poly a close second. We not only compared the popular tools and the state-of-the-art methods commonly used by surface physicists, but also included a deep learning model designed specifically for stripe noise removal. Some of these methods were considered to be the best methods for c-AFM image destriping at the time when LRR was not yet used in this field. All the results in this paper show that LRR is far better than all others at a noise level similar to the real noise and is also competitive at other noise levels.

Moreover, our designed noise model can simulate noisy images to enable quantitative image quality comparison. It is well matched to the real stripe noise, as confirmed by the visual and quantitative image quality comparisons.

Finally, the detailed methods overview and experimental results provide surface physicists with a clear comparison of 16 different destriping methods. This is important for c-AFM users for processing images to remove stripe noise. For other scanning probe modalities or any modality which is affected by stripe noise it may be useful as well.

In future work we will apply LRR in data processing pipelines, and explore its usefulness on other data.

## Data Availability

The ground truth image that supports the findings of this study is openly available in [DataverseNL] at https://doi.org/10.34894/OYIGPC, reference number [1110]. All the image data are also available in the comparison code which at https://zenodo.org/record/8336262.

## Code availability

All of the comparison code and demos with images (including our methods and other methods) that accompanies this work is freely available at https://zenodo.org/record/8336262. The comparison modules of the other methods were each rewritten from their open source codes: Median, Modus, MD, Matching, FLT, Poly, TM, TMD, RS. Code in Gwyddion can be found at http://gwyddion.net/documentation/head/pygwy/gwy-module.html. The WNN code can be found at https://github.com/jtguan/Wavelet-Deep-Neural-Network-for-Stripe-Noise-Removal. For the DS2 code, see http://biodev.cea.fr/destripe/. For the VSNR code, see https://www.math.univtoulouse.fr/~white/Codes/VSNR/VNSR_VariationalStationaryNoiseRemover.html.
